# Effect of decision to delivery interval on perinatal outcomes during emergency cesarean deliveries in Ethiopia: A prospective cohort study

**DOI:** 10.1371/journal.pone.0258742

**Published:** 2021-11-08

**Authors:** Tebabere Moltot Kitaw, Birhan Tsegaw Taye, Mesfin Tadese, Temesgen Getaneh

**Affiliations:** 1 Department of Midwifery, Debre Berhan University, Debre Berhan, Ethiopia; 2 Department of Midwifery, Debre Markos University, Debre Markos, Ethiopia; Universita degli Studi dell’Insubria, ITALY

## Abstract

**Background:**

The National guidelines of most developed countries suggest a target of 30 minutes of the decision to delivery interval for emergency cesarean section. Such guidelines may not be feasible in poorly resourced countries and busy obstetric settings. It is generally accepted that the decision to delivery interval should be kept to the minimum time achievable to prevent adverse outcomes. Therefore, this study aimed to determine the average decision to delivery interval and its effect on perinatal outcomes in emergency cesarean section.

**Methods:**

A prospective cohort study was conducted from May to July 2020 at Bahir Dar City Public Hospitals. A total of 182 participants were enrolled, and data were collected using a structured and pre-tested questionnaire. A systematic sampling technique was applied to select the study subjects. Data were cleaned and entered into Epi-Data version 4.6 and exported to SPSS version 25 software for analysis. Logistic regression analysis was performed to identify predictors of outcome variables, and variables with a p-value of <0.05 were considered statistically significant.

**Results:**

The average decision to delivery interval was 43.73 ±10.55 minutes. Anesthesia time [AOR = 2.1, 95%CI = (1.3–8.4)], and category of emergency cesarean section [AOR = 3, 95% CI = (2.1–11.5)] were predictors of decision to delivery interval. The prolonged decision to delivery interval had a statistically significant association with composite adverse perinatal outcomes (odds ratio [OR] = 1.8, 95% confidence interval [CI] = (1.2–6.5).

**Conclusion:**

The average decision to delivery interval was longer than the recommended time. It should always be considered an important factor contributing to perinatal outcomes. Therefore, to prevent neonatal morbidity and mortality, a time-dependent action is needed.

## Introduction

The cesarean section defines the birth of a fetus via laparotomy followed by hysterotomy [1]. If it is done for the immediate life-threatening condition of the fetus or mother, it is called emergency cesarean section (EmCS) [2]. National guidelines in the United States and the United kingdom suggest a target of 30 min decision to delivery interval (DDI) for EmCS [3,4]. However, such guidelines are not well evidenced [5] and may not be feasible in poorly resourced countries and busy obstetric settings [6]. Globally, it is generally accepted that the DDI should be kept to the minimum achievable time because preventing adverse perinatal outcomes is critically time-dependent [7], and also, failing to do so might result in medico-legal complications [6].

All facilities that provide comprehensive obstetric care should respond to obstetric emergencies within the time limit [4]. However, EmCS might take time to initiate in developing countries, and the resources needed may not be adequate. This means that the risks associated with EmCS could be much higher in developing nations than in developed nations [2]. In many developing countries, maternal and neonatal morbidity and mortality rates are high [8]. For example, in Ethiopia, the neonatal mortality rate is 29 deaths per 1,000 live births [9]. In particular, after EmCS, neonatal mortality and morbidity were high. In addition, up to 14% of newborn loss has been reported after delivery by EmCS [10]. Therefore, only access to EmCS is insufficient, and the quality of the service is necessary for a better perinatal outcome [3]. Thus, this study aimed to determine the average DDI of the selected obstetric units and to evaluate the effect of DDI on perinatal outcomes in EmCS.

## Materials and methods

### Study design, setting, and participants

A prospective cohort study was conducted from May 2020 to July 2020 at the Bahir Dar city public hospitals in the Amhara region. In Bahir Dar City, there are three public hospitals, all of which are included in the study. All hospitals provided all types of obstetric care, and each hospital had two operation tables for cesarean section. All women who underwent EmCS during the study period at Bahir Dar City Public Hospitals were the study population. In this study, women who underwent EmCS with a preterm fetus, uterine rupture before the decision, intrauterine fetal death, and fetuses with gross congenital anomalies were excluded.

### Study variable

Decision-to-delivery interval and perinatal outcomes were the dependent variables. Socio-demographic characteristics such as age, marital status, occupation status, place of residence, and educational status and obstetric factors such as gravidity, parity, antenatal follow up, danger sign for current pregnancy, referral status, an indication of EmCS, stage of labor at the decision, fetal weight, anesthesia time, and intraoperative difficulties were included as independent variables.

### Operational definition

#### Emergency cesarean section

It can be classified as category-1 (immediate threat to the life of the woman or fetus) and category-2 (maternal or fetal compromise, which is not immediately life-threatening) [4].

#### Decision-to-delivery interval

A time range from the decision for EmCS to delivery of a newborn. After calculating to the nearest minute, 30min was used as the cutoff point. More than 30 min were exposed, and 30 min and below min were the non-exposed group [3,11,12].

#### Anesthesia time

The time taken from arrival at operation theater to skin incision time and 10 minutes used as a cutoff point to say delayed or not [6].

#### Composite adverse perinatal outcome

Presence of one of the following perinatal outcomes: stillbirth, fifth minute APGAR score of less than seven, admission to the neonatal intensive care unit (NICU), and neonatal death [13].

### Sample size and sampling procedure

The sample size was computed using the Open Epi software (version 3.03 statistical software). The following assumptions were considered: proportion 12.3% [14], confidence level 95%, level of significance, α = 5%, power of the study (1-β), 80%, margin of error, d = 5%. By adding a 10% non-response rate, the final sample size was 183. The total sample size was proportionally allocated for all three hospitals. After selecting the first participant by the lottery method in each hospital, the other study participants were selected using a systematic random sampling technique with a sampling interval of two.

### Data collection methods and materials

Properly designed, structured, and interviewer-administered questionnaires and checklists were used to collect the data. The tool was prepared after reviewing related literature [6,13–17] and was modified and validated by experts to fit the local situation and research objectives (see S1 File). The study enrolled six BSc midwives and three general practitioners as data collectors and supervisors, respectively. An interviewer-administered questionnaire was used to collect socio-demographic characteristics and obstetric characteristics. Data were collected from the time of the decision to the time of discharge to home or death of neonates captured by an observational checklist. Before collecting the actual data, a pre-test was done on 10% of the samples, and training for data collectors and supervisors was provided to ensure data quality. The trained data collectors were supervised during data collection, and each questionnaire was checked for completeness daily.

### Data processing, analysis, and interpretation

Supervisors and principal investigator checked the data for its ‘completeness and consistency, then manually cleaned and coded, and entered into Epi-Data version 4.6, lastly exported to SPSS version 25 for analysis. As recommended by scholars, bivariate logistic regression analysis was done for each the independent variables. In the bivariate logistic regression analysis, variables with a p-value of ≤0.25 were candidates for the multivariable logistic regression model. The strength of association between independent and dependent variables was interpreted using an adjusted odds ratio (AOR) with a 95% confidence interval. The Hosmer-Lemeshow goodness-of-fit was applied to test for model fitness. Variables with a p-value <0.05 in the multivariable logistic regression model were considered statistically significant.

#### Ethical consideration

Ethical clearance was obtained from the Institutional Review Board (IRB) of Bahir Dar University College of Medicine and Health Science. In addition, official permission was obtained from all the hospital administrative bodies. Verbal consent preoperatively and then written consent after stabilization postoperatively was obtained from the individual participant. All the participants in the study participated voluntarily, and their information was kept confidential, and participants had the right to protest at any time.

## Results

### Socio-demographic characteristics

A total of 182 study subjects participated in this study, resulting in a 99.45% response rate. The median age of participants was 27 years, with an interquartile range (IQR) of 24 to 30 years. The majority of the respondents, 168 (92.3%), were married, and 66 (36.3%) had no formal education (Table 1).

**Table 1:** Sociodemographic characteristics of respondents, in Bahir Dar City Public Hospitals, Ethiopia, 2020 (n = 182).

Variable	Categories	n (%)
AGE in Years	<20	4(2.2)
	20–24	51(28)
	25–29	62(34)
	30–34	44(23.6)
	≥35	21(11.5)
Marital Status	Single	8(4.4)
	Married	168(92.3)
	Divorced	6(3.3)
Educational Status	No Formal Education	66(36.3)
	Primary School	43(23.6)
	Secondary School	30(16.5)
	Collage / university	43(23.6)
Occupational Status	Government Employee	26(14.3)
	House Wife	104(57.1)
	Daily Labor	21(11.5)
	Merchant	23(12.6)
	Others*	8(4.4)
Place of Residence	Urban	128(70.3)
	Rural	54(29.7)

Others*(student, nongovernmental organization employee).

### Obstetrics characteristics

About 81(44.5%) women became pregnant for the first time. Forty-two (23.1%) women were primiparous, and nearly two-thirds, 118 (64.7%) women, had four or more antenatal care during this pregnancy, whereas 11 (6%) women had no ANC follow-up. Of the women who received antenatal care, two-thirds were counseled about birth preparedness and complication readiness plans. Among women who faced danger signs during pregnancy, 42% were due to symptoms of preeclampsia (Table 2).

**Table 2:** Obstetrical characteristics of respondents in Bahir Dar City Public Hospitals, Ethiopia, 2020 (n = 182).

Characteristics	Categories	n(%)
Gravidity	Primigravidia	81(44.5)
	Multigravida	80(44)
	Grand multipara	21(11.5)
Parity	Nulliparous	85(46.7)
	Prim parous	42(23.1)
	Multiparous	43(23.6)
	Grand multiparous	12(6.6)
ANC follow up	First visit	5(2.7)
	Second visit	11(6)
	Third visit	37(20,3)
	Fourth and above	118(64.7)
	No ANC follow up	11(6)
Danger sign during this pregnancy	Vaginal bleeding	11(6)
	Blurred Vision/Severe Headache	14(7.6)
	Gush of fluid per vagina	5(2.7)
	Others*	3(1.6)
	No	149(81.9)
Referral status	Yes	122(67)
	No	60(33)

Others* (fever, offensive vaginal discharge, and breech at or above 36 weeks of gestation).

### Decision to delivery interval by indication

In the majority of cases, 150(82.4%), the EmCS was performed with a DDI of 31–75 minutes. Whereas, 26 (14.3%) and 6 (3.3%) of EmCS was performed within 30 minutes and >75 min of the decision to delivery interval, respectively. The mean ± SD (standard deviation) DDI observed in the study was 43.73 ±10.55 minutes. Thus, the meantime of category-1 emergency cesarean section was lower than that of category-2 by 7 minutes (Table 3).

**Table 3:** Indication for emergency cesarean section, in Bahir Dar City Public Hospitals, Ethiopia, 2020 (n = 182).

Category	Indication	DDI in min		Total	Mean ± SD min
		≤30	>30		
Category-1	Cord Prolapse	6	0	6	39.9±7.12
	APH (AP/PP)*	3	11	14	
	NRFHBP	8	42	50	
	Others**	4	6	10	
Category -2	CPD	0	20	20	46.75± 14.75
	Failed induction	0	9	9	
	Failed VBAC*** and >1 Scar at Labor	2	25	27	
	Breech	0	14	14	
	First stage labor disorders with meconium	3	29	32	

APH(AP/PP) * (APH = antepartum hemorrhage, AP = Aburatio placenta, Placenta Previa), Others** (severe preeclampsia, eclampsia, failed instrument), VBAC***(vaginal delivery after cesarean section).

### Predictors of the decision to delivery interval

From the five variables included in the multivariable logistic regression, the EmCS category [AOR = 3, 95% CI = (2.1–11.5)] and anesthesia time [AOR = 2.1, 95%CI = (1.3–8.4)], were predictors of the decision to the delivery interval. The analysis revealed that women whose anesthesia time was more than 10 min were two times as likely to have a delayed decision to delivery interval than women who were transferred at 10 min and less min. In addition, women whose EmCS was performed for category two EmCS indications were three times more likely to have a delayed decision to delivery interval than those in category one (Table 4).

**Table 4:** Predictors of decision to delivery time intervals, in Bahir Dar City Public Hospitals, Ethiopia, 2020 (n = 182).

Variables (reference)	Bivariable regression		Multivariable regression	
	P-value	COR(95%CI)	AOR(95%CI)	P-value
Stage of labor at decision, Second stage (First stage)	0.16	1.4(.32–6)	0.6(0.1–1.1)	0.81
Gestational Age, Unknown (Known)	0.22	1.07(0.4–2.8)	1.19(0.2–6.0)	0.92
New born birth weight(kg) ≥2.5 (<2.5)	0.09	1.2(0.5–4)	1.1(0.03–4.3)	0.1
Category of EmCS Category 2 (Category 1)	0.001	6.9(4–12.6)	3 (2.1–11.5)	**0.002**
Previous uterine scare present yes (no)	0.05	2.3(.5–10.0)	0.9(0.13–6.0)	0.90
Intraoperative difficulty no (yes)	0.094	0.38(0.13–1.1)	0.42(.1–1.6)	0.2
Anesthesia time >10 min (≤10 min)	0.003	3.6(1.5–8.5)	2.1(1.3–8.4)	**0.012**

### The decision to delivery interval and perinatal outcomes

**Fig 1:**
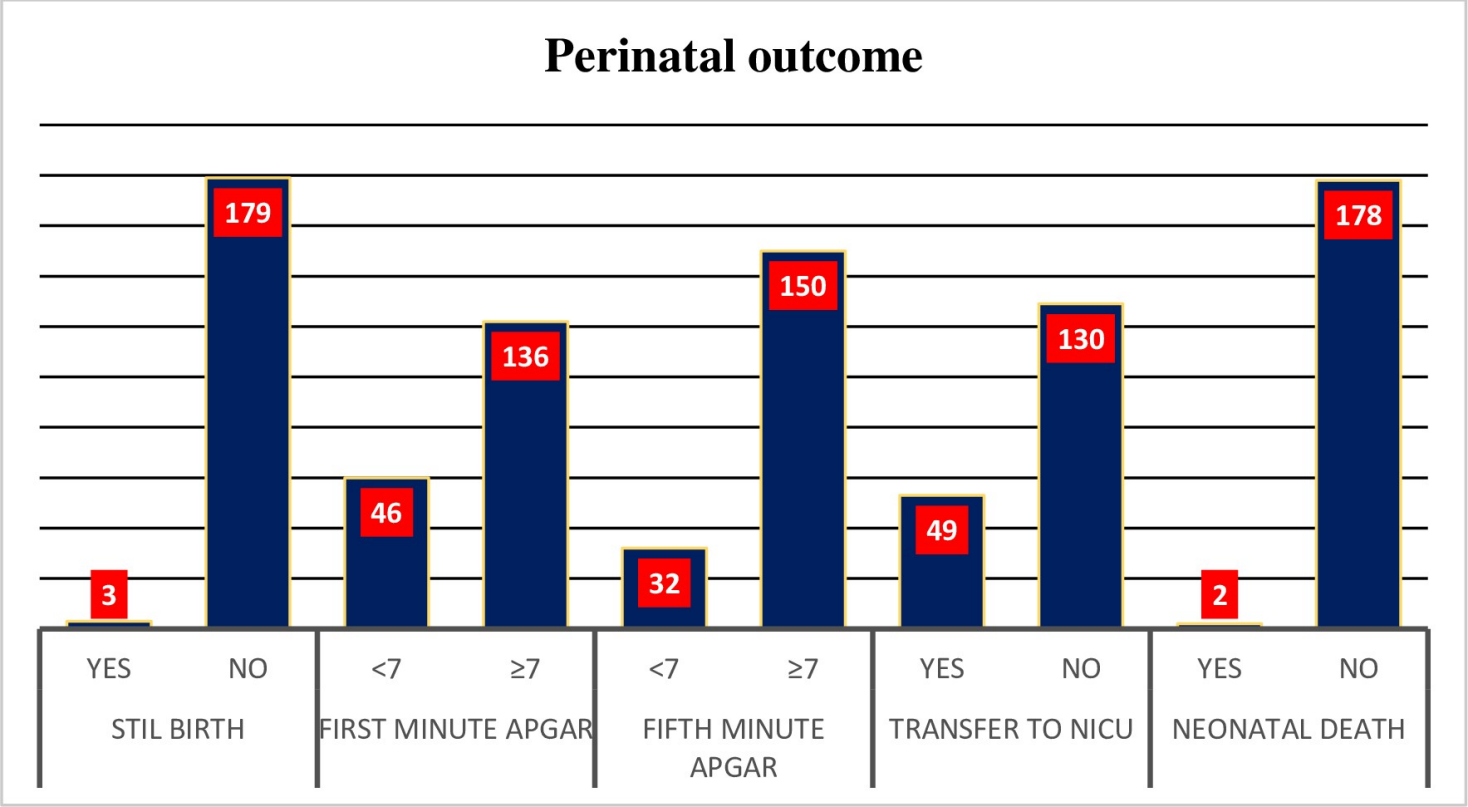
Shows perinatal outcomes of emergency caesarean section in Bahir Dar city public Hospitals Northwest Ethiopia, 2020 (n = 182).

From a total of 182 deliveries, three were alive fetuses when the decision for EmCS was performed but had no sign of life (stillbirth) at birth. The indication for EmCS with these stillbirths was cord prolapse and placental abruption. An Apgar score <7 was recorded in 46 newborns in the 1^st^ minute and 32 in the 5^th^ minute. Among forty-nine neonates who were transferred to the NICU, two died before discharge. Both dead neonates were delivered 30 min after admission (Fig 1).

### Effect of decision to delivery interval on perinatal outcome

Bivariate logistic regression analysis revealed that there was no statistically significant association between delayed DDI (>30 min) and individual adverse perinatal outcomes (neonatal admission, stillbirth, neonatal death until discharge, less than seven 1^st^ and 5^th^ minute Apgar scores). However, it is associated with adverse composite perinatal outcomes. This finding showed that a newborn who delivered after 30 minutes of decision had 1.8 times to have adverse perinatal outcomes when we compare with newborn delivered within 30 minutes of decision [OR = 1.8, 95% CI = (1.2–6.5) (Table 5).

**Table 5:** Bivariate logistic regression analyses results: DDI vs. perinatal outcome in emergency cesarean section in Bahir Dar City Public Hospitals, Ethiopia, 2020.

DDI	Prenatal Outcome		OR(95%CI)	P-value
	**Frist minute APGAR score**			
	<7 APGAR	≥7 APGAR		
≤30	6	20	1	
>30	40	116	1.15(0.4–3)	0.7
	**Fifth minute APGAR**			
	<7 APGAR	≥7 APGAR		
≤30	6	20	1	
>30	26	130	0.6(0.2–1.2)	0.42
	**Admission to NICU**			
	Yes	No		
≤30	5	20	1	
>30	44	110	1.6(0.5–4.5)	0.056
	**Adverse perinatal outcome**			
	Yes n(%)	No n(%)		
≤30	6	20	1	
>30	55	101	**1.8(1.2–6.5)**	**0.037**

## Discussion

This study found that 14.3% of women were operated within 30 minutes of decision to delivery interval with a mean ± SD DDI of 43.73±10.55 min. This is consistent with studies conducted in North and South Gondar, Ethiopia, in which the DDI within 30 min were 19.6 and 17.5%, respectively [12,18]. This study’s mean DDI also parallels the study finding in North Gondar Ethiopia, which was 42 ±21.4 [12]. The similarity may be due to similarities in the educational backgrounds of staff. In addition, constraints in surgical material and materials for EmCS preparation in the hospitals and similarity in administrative issues may also contribute to the consistency of the study findings since all of these public hospitals are under the federal minister of health.

On the other hand, studies conducted in Nigeria and Uganda showed that the meantime for DDI was 119.2 and 92 min, respectively [13,19]. This difference may be due to a lack of funds for surgical materials, and patients’ relatives usually pay surgical fees before the operation could be performed in the study area of Nigeria [19], which may prolong DDI in the area. Differences in Uganda’s study may be due to the data collection method, as Uganda data were collected from maternal records.

On the contrary, our result is lower than research conducted to evaluate the effect of a program to shorten the decision-to-delivery interval for emergent cesarean section on maternal and neonatal outcomes in Israel, with the mean DDI of 21.7 ± 9.1 min and 12.3_±3.8 min before and after program implementation [20]. The difference may be explained by the socioeconomic difference between these two study areas.

In this study, anesthesia time was positively associated with prolonged DDI during emergency cesarean section. This is consistent with research done in India [6,21]. This may be due to limitation of operation table, repeated trial for spinal anesthesia and may also be due to shortage of OR material. This may also be due to faulty equipment and ineffective coordination between obstetricians, anesthetics, and nurses. Women whose EmCS was performed for category two EmCS indications were three times more likely to have a delayed decion to delivery interval than those in category one. This is consistent with studies conducted in Uganda, India, and Saudi Arabia [6,13,22]. This may be due to the urgent response of providers for cases that are an immediate threat to the life of the woman or fetus than maternal or fetal compromise, which is not immediately life-threatening.

Decision-to-delivery interval is a critical period between the decision and delivery of the newborn because preventing adverse perinatal outcomes is critically time-dependent [7]. Our study showed no statistically significant association between delayed DDI (>30min) and neonatal admission, stillbirth, neonatal death until discharge, less than seven 1^st^ and 5^th^ minute Apgar scores. The result is similar to study findings in Ethiopia (South and North Gondar), Tanzania, India, and Nigeria [6,12,14,23]. But from our study area, three stillbirths and two neonatal losses until discharge were reported. This is similar to a report from India in which a similar number of stillbirths occurred while waiting for EmCS [21]. There may be several possible justifications, especially in resource-limited and busy obstetric units. From these: problem in early detection and treatment, absence of needed material on hand, busy and preoccupied operation table, and delay due to the mother’s hesitation for operation consent. In general, such poor outcomes indicate an inability to act urgently. On the other hand, a statistically positive association was observed between composite adverse perinatal outcomes and delayed DDI (AOR = 1.8,95% CI = (1.2–6.5)). This is comparable with the largest population-based study in Germany, which revealed an association between longer DDI and adverse perinatal outcomes [24]. Another study from Israel also reported a significant improvement in neonatal outcomes after DDI in EmCS was shortened [20].

Since the study is prospective which could make it appropriate to identify factors. The author acknowledged that the study assessed only immediate neonatal outcomes, having a small sample size and observer bias are limitations of the study.

## Conclusion

In this setting, the average decision to delivery interval was longer than the recommended time. A decision delivery interval should always be considered as one of the important contributing factors for adverse neonatal outcomes, especially in crash EmCS (cord prolapse and antepartum hemorrhage with active bleeding). Therefore, clinical judgment is required to assess the urgency of cesarean section to prevent neonatal morbidity and mortality.

## Supporting information

S1 File Questionniare. (DOCX)

S2 File Data set. (SAV)

## Abbreviations

AOR: adjusted odds ratio
CI: confidence interval
DDI: decision to delivery interval
EmCS: emergency cesarean section
NICU: neonatal intensive care unit
SD: standard deviation
