# Supplementary material for: Effect of decision to delivery interval on perinatal outcomes during emergency cesarean deliveries in Ethiopia: A prospective cohort study
Source: PLoS One. 2021 Nov 8;16(11):e0258742. doi: 10.1371/journal.pone.0258742 (PMC8575252; doi:10.1371/journal.pone.0258742)
Supplement: S1 File — (DOCX) [file pone.0258742.s001.docx]

**English version questionnaire**

|  | **Part I: Question on socio-demographic characteristics** | | | |
| --- | --- | --- | --- | --- |
| S.R | Question | Possible answer | Code | Skip |
| 101 | Age in yrs |  |  |  |
| 102 | Marital Status | Single | 1 |  |
|  |  | Married | 2 |  |
|  |  | Divorced | 3 |  |
|  |  | Widowed | 4 |  |
| 103 | Educational status | Can't read and write | 1 |  |
|  |  | Read and write | 2 |  |
|  |  | Elementary | 3 |  |
|  |  | High school | 4 |  |
|  |  | Preparatory | 5 |  |
|  |  | Diploma and above | 6 |  |
| 104 | Occupation | House wife | 1 |  |
|  |  | Government Employee | 2 |  |
|  |  | Daily labor | 3 |  |
|  |  | Farmer | 4 |  |
|  |  | Merchant | 5 |  |
|  |  | Other(specify)______________ | 6 |  |
| 105 | Place of residence | Urban | 1 |  |
|  |  | Rural | 2 |  |

| **Part II: Obstetrics related questions** | | | | |
| --- | --- | --- | --- | --- |
| 201 | Number of pregnancy |  |  |  |
| 202 | Number of delivery |  |  |  |
| 203 | Number of alive children | ___________ |  |  |
| 204 | Did you have ANC follow-up in your recent pregnancy? | Yes | 1 | If no to Q#207 |
|  |  | No | 2 |  |
| 205 | How many times did you visited ANC clinic? | One | 1 |  |
|  |  | Two | 2 |  |
|  |  | Three | 3 |  |
|  |  | Four and above | 4 |  |
| 206 | Did you counseled on BPCR plan during ANC follow up. | Yes | 1 |  |
|  |  | No | 2 |  |
| 207 | Do you have danger sign during this pregnancy | Yes | 1 | If no to Q#209 |
|  |  | No | 2 |  |
| 208 | If yes for Q#207 which type of danger sign | Vaginal bleeding | 1 |  |
|  |  | Blurred vision | 2 |  |
|  |  | Severe headache | 3 |  |
|  |  | Gush of fluid per vagina | 4 |  |
|  |  | Other (specify)_____ | 5 |  |
| 209 | Gestational age in Wks (from reliable LNMP or early US) | If Known _______wk | 1 |  |
|  |  | Unknown | 2 |  |
| 210 | Are you referral | Yes | 1 |  |
|  |  | No | 2 |  |
| 211 | Do you have history of delivery by EmCS( Only for parous women) | Yes | 1 |  |
|  |  | No | 2 |  |

| **Part III: Decision to delivery interval related questions** | | | | | | | | | | |  |  |
| --- | --- | --- | --- | --- | --- | --- | --- | --- | --- | --- | --- | --- |
| S.N | Question | | | | Possible answer | | Cod | | Skip | |  |  |
| 301 | Cervical dilation at decision for EmCS | | | |  | |  | |  | |  |  |
| 302 | Indication for cesarean section( if there is more than one take the most emergent) | | | | ––––––––––––––––––––––––––– | |  | |  | |  |  |
| **Fill by Observation** | | | | | | | | | | | |  |
| 303 | | | Time at decision for emergency cesarean section | |  | |  | |  | | |  |
| 304 | | | Time while women arrival at Operation room | |  | |  | |  | | |  |
| 305 | | | OR material prepared while women arrive at OR | | Yes | |  | |  | | |  |
|  |  |  |  |  | No | |  | |  | | |  |
| 306 | | | Free, functional OR table present | | Yes | |  | |  | | |  |
|  |  |  |  |  | No | |  | |  | | |  |
| 307 | | | Anesthesia induction time | |  | |  | |  | | |  |
| 308 | | | No attempt for regional anesthesia | |  | |  | |  | | |  |
| 309 | | | Incision starting time | | ___________________ | |  | |  | | |  |
| 310 | | | Intra operative difficulty | | Yes(specify)------------------ | | 1 | |  | | |  |
|  |  |  |  |  | No | | 2 | |  |  |  |  |
| 311 | | | Baby out time | |  | |  | |  | | |  |
| 312 | | | End of surgery | |  | |  | |  | | |  |
| **Fetal outcome** | | | | | | | | | | | | |
| 313 | | Still birth | | Yes | | | | 1 | |  | | |
|  |  |  |  | No | | | | 2 | |  |  |  |
| 314 | | APGAR Score | | 1^st^ minute | |  | |  | |  | | |
|  |  |  |  | 5^th^ minute | |  | |  | |  |  |  |
| 315 | | Neonatal/ still birth weight | | ____________kgs | | | | | |  | | |
| 316 | | Is the neonate admitted to NICU | | Yes | | | | 1 | |  | | |
|  |  |  |  | No | | | | 2 | |  |  |  |
| 317 | | If Q#316 is yes admission diagnosis | |  | | | |  | |  | | |
| 318 | | Final out come | | Discharge alive | | | | 1 | |  |  |  |
|  |  |  |  | Dead | | | | 2 | |  |  |  |
| 319 | | If dead cause of death | |  | | | |  | |  | | |

**Thank You!!!**

የአማርኛ ቃለ መጠይቅ

ክፍል አንድ፡ ማህበራዊ ዳራን የሚዳስሱ ጥያቄዎች

| ተ.ቁ | መጠይቆች | አማራጭ መልሶች | መለያ ቁጥር | ምርመራ |
| --- | --- | --- | --- | --- |
| 101 | እድሜሸ ስንት ነው | ____________ ዓመት |  |  |
| 102 | የጋብቻ ሁኔታ | ያላግባች | 1 |  |
|  |  | ያገባች/ባለትዳር/ | 2 |  |
|  |  | የፈታች | 3 |  |
|  |  | ባሏ የሞተባት | 4 |  |
| 103 | የትምህርት ደረጃሽ | አልተማርኩም | 1 |  |
|  |  | መጻፍና ማንበብ እችላለሁ | 2 |  |
|  |  | 1ኛደረጃ | 3 |  |
|  |  | መለስተኛ ሁለተኛ ደረጃ | 4 |  |
|  |  | ፕሪፓራቶሪይ | 5 |  |
|  |  | ከኮልጅ በላይ | 6 |  |
| 104 | ስራሽ ምንድን ነው? | የቤት እመቤት | 1 |  |
|  |  | መንግስት ሠራተኛ | 2 |  |
|  |  | የቀን ሠራተኛ | 3 |  |
|  |  | ገበሬ | 4 |  |
|  |  | ነጋዴ | 5 |  |
|  |  | ሌላ ካለ---- | 6 |  |
| 105 | የት ነው የምትኖሪ? | ከተማ | 1 |  |
|  |  | ገጠር | 2 |  |

**ክፍል ሁለት: የሥነ-ተዋልዶ ጤናን በተመለከተ ለጥናቱ ተሳታፊዎች የተዘጋጀ መጠይቅ**

| ተ.ቁ | መጠይቆች | አማራጭ መልሶች | መለያ | ምርመራ |
| --- | --- | --- | --- | --- |
| 201 | ስንተኛ አርግዝናሽ ነዉ? | ______________ |  |  |
| 202 | ስንት ልጆች ወልደሻል? | ______________ |  |  |
| 203 | ስንት ልጆች በህይዎት አሉሽ? | ________________ |  |  |
| 204 | በእርግዝናሽ ሰዓት የነፍሰጡር ክትትል ነበረሽ? | አዎን | 1 | የለኝም ከሆነ #207 |
|  |  | የለኝም | 2 |  |
| 205 | ስንት ጊዜ የነፍሰጡር ክትትል አድርገሻል? | አንድ ጊዜ | 1 |  |
|  |  | ሁለት ጊዜ | 2 |  |
|  |  | ሦስት ጊዜ | 3 |  |
|  |  | አራት ጊዜ ና ከዚያ በላይ | 4 |  |
| 206 | በነፍሰጡር ክትትልሽ ወቅት ስለ ወሊድ ቅድመ ዝግጅት እና ሊከሰቱ ስልሚችሉ አደገኛና ውስብስብ ችግሮች ምክር አግኝተሻል | አዎን | 1 |  |
|  |  | የለም | 2 |  |
| 207 | በነፍሰጡርነትሽ ወቅት ያጋጠመሽ ችግር ነበር? | አዎን | 1 | የለም ከሆነ ወደ ጥ#209 |
|  |  | የለም | 2 |  |
| 208 | ለጥያቄ # 207 አዎን ከሆነ መልስሸ ያገጠመሽ ችግር ምን ነበር? | የደም መፍሰስ ችግር | 1 |  |
|  |  | የዓይን ብዥታ | 2 |  |
|  |  | ከፍተኛ የሆነ ራስ ምታት | 3 |  |
|  |  | የእንሽርት ውኃ ቀድሞ መፍሰስ ችግር | 4 |  |
|  |  | ሌላ ችግር(ካለ ይጻፉ)--------- | 5 |  |
| 209 | የርግዝና ወቅት በሳምንት ሲሰላ( ከመጨረሻ የወር አበባ/ከ ቀደመ አልትራሳውነድ/ | የሚታወቅ ከሆነ ---------ሳምንት ነው | 1 |  |
|  |  | አይታወቅም | 2 |  |
| 210 | ሪፈር ተብለሽ ነው? | አዎን | 1 |  |
|  |  | አይደለም | 2 |  |
| 211 | ከዚህ በፊት በኦፕሬሽን ወልደዋል | አዎ | 1 |  |
|  |  | አልወለድኩም | 2 |  |

| **ክፍል ሦሥት፡ ኦፕሬሽን ለመስራት ከተወሰነበት ልጁ እስከተወለደበት ስላልዉ ጊዜ የሚያጠነጥኑ መጠየቆች** | | | | | | | |
| --- | --- | --- | --- | --- | --- | --- | --- |
| S.No | ጥያቄ | የምላሽ አማራጮች | | መለ | ምርመራ | | |
| 301 | ኦፕሬሽን ለመስራት ሲወሰን የማህጸን በር/ ጫፍ/ምን ያክል ከፍቷል /ስፋት |  |  | |  | | |
| 302 | ኦፕሬሽኑ የተሰራብት ምክንያት ምንዲን ነው (ከ አንድ በላይ ከሆነ በጣም አስቸኳዩን ውሰድ/ጅ) |  |  | |  | | |
| **በምልከታ የሚሞላ መጠይቅ** | | |  | |  | | |
| 303 | ኦፕሬሽን ለመስራት የተወሰነበት ሰዓት |  |  | |  | | |
| 304 | ኦፕራሽን ክፍል የደረሰችበት ሰዓት |  | |  | |  |  |
| 305 | ኦፐሬሸን ክፍል ስትደርስ የኦአር መሳሪያዎች ተዘጋጅተዋል ወይ | አዎ | | 1 | |  |  |
|  |  | የለም | | 2 | |  |  |
| 307 | ስራ ላይ ያልሆነ ኦአር ቴብል አለ | አዎ | | 1 | |  |  |
|  |  | የለም | | 2 | |  |  |
| 308 | የስመመን መድሀኒት መሰጠት የጀመረቺበት ሰዓት |  | |  |  | | |
| 309 | የወሰደቺው የማደንዘዣ / ስመመን / አይነት | ጀኔራል | | 1 |  | | |
|  |  | ሪጅናል | | 2 |  |  |  |
| 310 | ሪጅናል ከሆነ በስንት ሙከራ ተሳካ |  | |  |  | | |
| 311 | ኦፕሬሽኑ የተጀመረበት ሰዓት |  | |  |  | | |
| 312 | በኦፕሬሽን ሰዐት የገጠመ አስቸጋሪ ሁኔታ አለ? | አዎን(ምንነበር?)__________ | | 1 |  | | |
|  |  | የለም | | 2 |  |  |  |
| 313 | ልጁ/ዋ የተወለደበት/ችበት ሰዓት |  | |  |  | | |

| **314** | **የህጻኑ/ኗ ሁኔታ** | | | | | |
| --- | --- | --- | --- | --- | --- | --- |
| 315 | ሞቶ/ታ ነው የተወለደው/ቺው | አዎን(ምክንያቱ) | | 1 |  |  |
|  |  | አይደለም | | 2 |  |  |
| 316 | የአፒጋር ውጤት | 1ኛ ደ |  |  |  |  |
|  |  | 5ተኛ ደ |  |  |  |  |
| 317 | የህጻኑ/ዋ ክብደት | ---------------ኪሎግራም | |  |  |  |
| 318 | ህጻኑ/ዋ ጨቅላ ህጻናት ህክምና ክፍል ተኝቷል /ታለቺ | አዎ | |  |  |  |
|  |  | አይደለም | |  |  |  |
| 319 | ጥያቄ #318 አዎን ከሆነ የገባበት/የገባቺበት ምክንያት ምን ነበር |  | |  |  |  |
| 329 | በህይወት ከተወለደ/ች በኋላ ህይወት ማለፍ አለ | አዎን(ምክንያቱ ምን ነበር) | |  |  |  |
|  |  | የለም | |  |  |  |

**መጠይቁ ተጠናቋል!!! አመሰግናለሁ!!!**
